# Neuroinflammatory Basis of Depression: Learning From Experimental Models

**DOI:** 10.3389/fncel.2021.691067

**Published:** 2021-07-02

**Authors:** Ruqayya Afridi, Kyoungho Suk

**Affiliations:** ^1^BK21 Plus KNU Biomedical Convergence Program, Department of Pharmacology, School of Medicine, Kyungpook National University, Daegu, South Korea; ^2^Brain Science and Engineering Institute, Kyungpook National University, Daegu, South Korea

**Keywords:** glia, depression, neuroinflammation, cytokines, immune cells, experimental models

## Abstract

The neuroinflammatory basis of depression encompasses the detrimental role of otherwise supportive non-neuronal cells and neuroinflammation in hampering neuronal function, leading to depressive behavior. Animals subjected to different stress paradigms show glial cell activation and a surge in proinflammatory cytokines in various brain regions. The concept of sterile inflammation observed in animal models of depression has intrigued many researchers to determine the possible triggers of central immune cell activation. Notably, microglial activation and subsequent phenotypic polarization in depression have been strongly advocated by the wealth of recent preclinical studies; however, findings from human studies have shown contradictory results. Despite intensive investigation, many research gaps still exist to elucidate the molecular mechanisms of neuroinflammatory cascades underlying the pathophysiology of depression. In this mini-review, recent progress in understanding neuroinflammatory mechanisms in light of experimental models of depression will be thoroughly discussed. The challenges of mirroring depression in animal and *in vitro* models will also be highlighted. Furthermore, prospects of targeting neuroinflammation to treat depressive disorder will be covered.

## Introduction

Major depressive disorder (MDD) is the most prevalent debilitating psychiatric disorder affecting individuals during some part of their life, resulting in a substantial health and economic burden worldwide (Konig et al., 2019; Konnopka and Konig, 2020). Neurochemical and structural alterations in mesolimbic and corticolimbic neural circuitry that regulate mood and behavior, including the prefrontal cortex (PFC), amygdala, nucleus accumbens, and hippocampus, are reported to be the cause of depression symptoms (Duman et al., 2016). Dysregulation of monoaminergic neurotransmission, including serotonin and dopamine, is a widely accepted theory of depression pathology, and various perturbations in monoamine signaling and metabolism have been identified (Jesulola et al., 2018). Moreover, deficits in synaptic plasticity induced by altered glutamatergic neurotransmission are involved in depression pathology, constituting the “neuroplasticity” hypothesis of depression (Pittenger and Duman, 2008). Various drugs interfering with glutamatergic neurotransmission have been reported to exert antidepressant actions in clinical and preclinical studies (Pittenger and Duman, 2008). Brain-derived neurotrophic factor (BDNF) is known to play an important role in neuroplasticity, and decreased BDNF expression has been reported in various brain regions of depressed patients (Dwivedi, 2009). All of these mechanisms play a crucial role in the pathology of depression; however, the inefficacy of antidepressant drugs in a subpopulation of patients with MDD and decreased remission rates highlight the involvement of diverse mechanisms in addition to these neurocentric theories.

Emerging evidence provides ample support for the involvement of non-neuronal cells leading to a neuroin-flammatory milieu in depression neurobiology (Koo and Duman, 2008; Steiner et al., 2011; Strawbridge et al., 2015). Glial cells constitute a major proportion of brain tissue and play a significant role in maintaining brain homeostasis by supporting neurons in dynamic ways. Increased microglial inflammatory activation, astrocytic atrophy, and decreased myelin basic protein immunoreactivity and fewer mature oligodendrocytes have been documented in MDD subjects and animal models of depression (Cotter et al., 2001; Tynan et al., 2013; Yang et al., 2015). The inflammatory activation of microglial cells has been reported to alter glutamatergic neurotransmission, impair monoamine synthesis, and interfere with BDNF signaling, culminating in altered synaptic plasticity and neurogenesis, and precipitating depression (Weber et al., 2019).

Neuroinflammatory perturbations identified in animal models of depression provide a strong basis for non-neuronal cell involvement in MDD pathology (Weber et al., 2017; Wang et al., 2018). Several *in vitro* experimental models of MDD, which provide cell-level information, have been developed to enhance the usefulness of *in vivo* models (Zunszain et al., 2012; Zhang et al., 2020b). All these models are of value for deciphering the fundamental mechanisms underlying MDD pathology and testing novel therapies targeted against this disease. In this review, recent literature documenting neuroinflammatory alterations observed in experimental models of depression is discussed. Subsequently, plausible reasons behind discrepancies between data from human studies and preclinical data are highlighted. Additionally, the therapeutic significance of targeting neuroinflammation in depressive disorders will be discussed.

## Neuroinflammation in the Pathophysiology of Depression

Environmental and genetic factors have been identified as crucial drivers of depression pathology in both human and rodent models (Lesch, 2004; Levinson, 2006). As these factors are highly variable, epistatic, and complex, they are thought to regulate vulnerability to depression development and responsiveness to antidepressant therapy. Various polymorphisms have been reported in genes regulating the hypothalamic–pituitary–adrenal (HPA) axis, serotonin recycling, and immune responses, including corticotropin-releasing hormone receptor 1, the sodium-dependent serotonin transporter gene (*SLC6A4*), and interleukin-1β *(IL-1β)* (Baune et al., 2010; Schiele et al., 2021). Moreover, environmental stressors are associated with epigenetic modification of BDNF, its receptor tropomyosin-related kinase B gene, glucocorticoid receptor gene (*NR3C1*), and glutamate ionotropic receptor NMDA type subunit 2B *(GRIN2B)* (Ernst et al., 2009; Jiang et al., 2010; Sun et al., 2013; Efstathopoulos et al., 2018).

Accumulating evidence suggests the involvement of multiple biological systems, including the neuroendocrine system, immune system, and neural circuitry, in the pathophysiology of depression (de Kloet et al., 2005). Activation of the HPA axis results in increased cortisol secretion in the blood, which in turn activates peripheral immune cells (Otte et al., 2016). Inflammatory signals from peripheral immune cells are propagated through various humoral, neural, and cellular pathways and results in the activation of brain resident immune cells that interfere with neurotransmitters and directly affect neuronal integrity through excitotoxicity.

### Triggers and Mediators of Neuroinflammation in Depression

The activation of the HPA axis and sympathetic system is an adaptive response of an organism toward any psychological or environmental stimuli perceived as a threat, resulting in the release of glucocorticoids (GC) and norepinephrine (NE) in the blood (Selye, 1976; McEwen et al., 2016). Increased GC and sympathetic signaling exert proinflammatory effects by mobilizing immune cells from the bone marrow, lymph node, and spleen and increasing their inflammatory activation (Engler et al., 2004; Dhabhar et al., 2012; Powell et al., 2013). Inflammatory activation of monocytes and macrophages leads to increased secretion of proinflammatory mediators, including tumor necrosis factor-α (TNF-α), IL-1β, and interleukin-6 (IL-6) (Serrats et al., 2010). Increased proinflammatory cytokines in circulation also have the propensity to repress the expression of several tight-junction proteins of the blood–brain barrier (BBB), including claudin-5 (Dudek et al., 2020). Mice exposed to chronic social defeat stress (CSDS) exhibited decreased expression of claudin-5, positively correlating with heightened peripheral TNF-α in circulation (Dudek et al., 2020). Chronic stress-induced BBB leakage in an animal model of depression allows the passage of proinflammatory mediators (Menard et al., 2017).

Recent literature also highlights the potential role of gut microbiome in precipitating inflammatory signals in depression pathology. Among divergent pathways through which gut microbiota can alter behavior, leading to depressive-like outcomes, is an inflammation-to-brain mechanism (Guo et al., 2019). A study in rodents demonstrated the activation of the master regulator of the inflammatory pathway nuclear factor-κB (NF-κB) when gut microbiota was altered by chronic restraint stress (CRS). The depletion of Lactobacillus was accompanied by increased inflammatory cytokines as well as increased microglial activation in the hippocampus (Guo et al., 2019). Although the mechanism of immune cell activation was not investigated, it is quite plausible that inflammatory signaling is involved, as Lactobacillus treatment reduced inflammation and alleviated depressive symptoms in mice subjected to CRS, highlighting the potential role of the gut-inflammatory pathway in exerting behavioral consequences.

### Contribution of Malfunctioning Glia

Astrocytes, microglia, and oligodendrocytes are the major types of the glial population, each having a distinct role in healthy and diseased states. Accumulating evidence suggests that gliosis and inflammation lead to increased levels of proinflammatory cytokines and reactive oxygen species (ROS) in various brain regions, thereby contributing to neuronal damage and leading to altered mood and behavior. Astrocytes are the most abundant glial cells that provide metabolic and trophic support to neurons. Atrophy and reduction in number of astrocytes as well as a reduction in various astrocytic proteins have been documented in MDD pathology (Fatemi et al., 2004; Zhao et al., 2018). Reduced astrocyte numbers in the hippocampus, amygdala, and prefrontal cortex of MDD patients have been reported (Altshuler et al., 2010; Cobb et al., 2016). The excitotoxicity observed in MDD can be correlated with astrocytic dysfunction. The inability of astrocytes to uptake glutamate from the synaptic cleft leads to prolonged synaptic activation, which in turn leads to excitotoxicity (Choudary et al., 2005).

Microglia are highly plastic, brain resident macrophages, that mainly guard the brain parenchyma in addition to playing other physiological roles. Microglia display extensive phenotypic plasticity dependent on surrounding cues. Recent studies suggest the important role of stress-induced damage-associated molecular patterns as a primary signal in activating microglia. The primed state of microglia that is characterized by an increased expression of proinflammatory cytokines increases the propensity for the development of severe depressive symptoms (Wohleb et al., 2014). In the CSDS model, it has been reported that microglia-secreted proinflammatory cytokines are crucial for the recruitment of peripheral immune cells in stress-responsive brain regions, and these cells remain sensitized for a longer period after cessation of the acute stressful stimuli (Wohleb et al., 2014). In addition, the study provided useful insights into the temporal effects of stress on the neuroimmune axis (Wohleb et al., 2014). A surge in proinflammatory cytokines due to microglial activation and peripheral immune cell infiltration leads to the upregulation of microglial indolamine 2,3 dioxygenase (IDO) activity (Corona et al., 2013). Increased microglial IDO activity diverts tryptophan metabolism from serotonin to quinolinic acid (QUIN), which is a N-methyl-D aspartate receptor agonist, serving as a link between immune and neurotransmitter changes in depression. Increased inflammatory cytokines, including TNF-α, in microglial cells can also influence the neuronal re-uptake of monoamine neurotransmitters by regulating neuronal mitogen-activated protein kinase (MAPK), leading to an increased surface expression of monoamine transporters on neurons (Zhu et al., 2010). Cytokine-mediated increases in microglial QUIN and reduction in astrocytic glutamate uptake can lead to excessive glutamate levels and actions, thereby altering synaptic plasticity.

## Experimental Models of Depression

With advances in our understanding of molecular mechanisms of depression, efforts have been made to establish *in vivo* and *in vitro* models that can be used efficiently for a better understanding of the enigmatic pathophysiology of depression. Although still not completely achieved, few experimental models, including *in vivo* and *in vitro*, have been used frequently in neuroscience research.

### *In vivo* Models of Depression

Considering stress as a major factor in predisposing humans to the development of depression, most animal models used in preclinical studies are based on stress. Though many of these models lack etiological relevance, the hyperactive HPA axis, impaired neuroplasticity and neurogenesis, and altered neurotransmitters are consistent features of these models that can be paralleled with human depression disease. Thus, the contribution of these models in providing novel insights into depression pathology cannot be underscored. Specifically, the role of neuroinflammation in the pathophysiology of depression has been well-established in these models and explains the antidepressant action of certain anti-inflammatory drugs (Table 1).

**TABLE 1:** Neuroinflammatory markers in animal models of depression.

**Models**	**Markers of neuroinflammation**	**Brain regions**	**Impact on neurons**	**Comments**	**References**
CSDS	Increased IL-1β, IL-18, IL-6, and TNF-α in brain Increased microglial activation	Hippocampus Prefrontal cortex	Not discussed	Modulation of microglial activation state could be used as a therapeutic strategy to treat depressive disorders	Gu et al., 2021
CSDS	Increased mRNA of IL-1β and CCL2 in the hippocampus Increased microglial inflammatory activation	Hippocampus	Increased ΔFosB expression	IL-1 receptor pathway played a crucial role in mediating stress-induced depressive effects	DiSabato et al., 2020
CSDS CUMS CRS	Increased IL-1β, IL-18, IL-6, and TNF-α	Hippocampus	Not discussed	Increased NLRP1-inflammasome pathway was critical for the development of depression	Song et al., 2020
CRS	Increased IL-1β and caspase-1 expression	Prefrontal cortex	Not discussed	Neuroinflammatory cascades in the frontal cortex were crucial for driving depressive behaviors	MacDowell et al., 2021
CSDS CUMS CRS	Increased IL-1β and caspase-1 expression	Hippocampus	Reduced density of presynaptic proteins Impaired synaptic plasticity Altered glutamatergic neurotransmission	Caspase-1-mediated neuroinflammatory pathway impaired glutamatergic pathway leading to depression	Li et al., 2018
CRS	Increased reactive oxygen species Increased microglial inflammatory activation Increased IL-1β, IL-18	Hippocampus	Morphological changes in hippocampal neurons including enlarged pericellular spaces and irregular arrangement	Microglial GR-NF-κB-NLRP3 signaling induced depressive-like behaviors in mice	Feng et al., 2019
CUMS	Increased IL-1β and TNF-α Increased microglial activation	Prefrontal cortex Hippocampus	Neuronal dystrophy Reduced dendritic spine density	Microglial mediated neuronal remodeling induced behavioral despair and cognitive impairments	Horchar and Wohleb, 2019
CUMS	Increased IL-1β and TNF-α Increased microglial activation	Hippocampus	Neuronal atrophy Reduced dendritic spine density	Exaggerated inflammatory response in the hippocampus following exposure to stress	Xu et al., 2021
CUMS	Increased Iba-1 reactivity in stress-responsive regions Increased immune cell density in brain	Hippocampus	Reduced hippocampal neurogenesis	Targeting microglial inflammatory activation rescued stress-induced depression	Troubat et al., 2021

CSDS, chronic unpredictable mild stress (CUMS), and CRS are the most widely employed animal models to decipher the neuropathological basis of depression. Increased neuroinflammatory profile characterized by elevated cytokines and the C-C Motif Chemokine ligand 2 (CCL2), and reduced anti-inflammatory regulation of neuronal-derived fractalkine ligand (CX3CL1) and microglial receptor (CX3CR1) are shared features in these models (Wohleb et al., 2014; Ramirez et al., 2016). Increased proliferation of inflammatory microglia with concomitant increase of Iba-1 immunoreactivity in the hippocampal tissue of mice subjected to CRS and CUMS has been reported (Feng et al., 2019; Horchar and Wohleb, 2019). Mice subjected to CSDS stress also exhibited microglial activation, recruitment of peripheral macrophages into the brain, and anxiety-like behavior (Tang et al., 2018). Inflammatory activation of microglia, stimulation of the microglial NLRP [NLR (nucleotide-binding domain and leucine-rich repeat) family, pyrin domain containing] inflammasome, and increased IL-1β production in the PFC were demonstrated in CUMS (Pan et al., 2014). Microglia isolated from mice subjected to CSDS have gene ontology profiles signifying increased inflammation, phagocytosis, and ROS production (Lehmann et al., 2018). The behavioral phenotypes observed in these models, including anhedonia, decreased sociability, and despair, are positively correlated with inflammatory activation of microglia.

Deficits in synaptic plasticity, altered dendritic spine density, and impaired neurogenesis because of heightened neuroinflammation have been reported in CSDS, CRS, and CUMS. Increased caspase-1 signaling in hippocampal region of mice after CSDS, CRS, and CUMS leads to dysregulated glutamatergic neurotransmission accompanied by altered dendritic spine density and reduced synaptic plasticity (Li et al., 2018). The genetic and pharmacological targeting of the IL-1β-caspase-1 pathway rescued the development of depressive behaviors in mice, highlighting the crucial role of neuroinflammatory pathways in impairing neuronal integrity (Li et al., 2018). Microglia-derived IL-1 can exert its detrimental effects on neurogenesis indirectly by stimulating the HPA axis as well as directly by activating IL-1 receptors expressed on hippocampal neural progenitor cells, resulting in decreased cell proliferation that is mediated by the NF-κB signaling pathway (Koo and Duman, 2008). Hence, microglial inflammatory activation as well as the neuroinflammatory milieu in animal models of depression may play key roles in the pathophysiology of depression.

### *In vitro* Models of Depression

Depression research is hampered by the absence of *in vitro* models that can recapitulate all molecular mechanisms of the disease. Attempts to model depression *in vitro* using hippocampal progenitor cell lines (HPCs) to study the pathways causing impaired neurogenesis are emerging. Thus far, studies have focused on isolated cell types in culture or occasionally two cell types in co-culture, which cannot fully model the important contributions of various cell types in disease. Various depressogenic stimuli identified in clinical and preclinical studies are used to study the mechanism or unravel pharmacological targets in neuronal cells and glial cell cultures (Table 2). Neurogenesis in the hippocampus regulates the HPA axis via a negative feedback mechanism; hence, the mechanisms underlying impairments in adult hippocampal neurogenesis have been explored *in vitro* (Schloesser et al., 2009). Microglia isolated from the hippocampus of cytokine-induced depressed mice suppressed neural stem/precursor cell proliferation and stimulated apoptosis of immature neurons, highlighting the role of microglia in impairing neurogenesis in depression pathology (Zhang et al., 2020a). IL-1β inhibited neurogenesis in HPCs by activating the neurotoxic branch of the kynurenine pathway, which has been postulated to be involved in the development of depressive disorders (Zunszain et al., 2012).

**TABLE 2:** Experimental findings in *in vitro* model of depression.

**Study type**	***In vitro* models**	**Depressogenic stimuli**	**Outcome measured**	**Comments**	**References**
**Mechanistic**	HPCs	IL-1β	Neurogenesis	IL-1β impaired neurogenesis by activating the neurotoxic kynurenine pathway, which has been implicated in depression pathology	Zunszain et al., 2012
	HPCs	Cortisol	Neurogenesis	Cortisol impaired neurogenesis in Serum/Glucocorticoid Regulated Kinase 1-dependent manner	Anacker et al., 2013
	HPCs	IL-1β and IL-6	Neurogenesis	IL-6 impacted neurogenesis in a concentration-dependent manner	Borsini et al., 2020
	Mouse primary microglial cells BV-2 mouse microglia cells	LPS	Microglial morphological changes	Circular RNA DYM was crucial for suppressing microglial activation, which was found to be decreased in MDD patients and *in vivo* models of depression	Zhang et al., 2020b
	HT-22 mouse hippocampal neuronal cells	Corticosterone	Cell proliferation	microRNAs rescues corticosterone induced impaired neurogenesis by inhibiting Sgk1	Jin et al., 2016
	Co-culture of primary microglial cells and NPSCs obtained from mice	Primed microglia isolated from IFN-γ-injected mice	Neuronal proliferation	Impaired neurogenesis has been associated with depression, and microglial inflammatory activation played a crucial role	Zhang et al., 2020a
**Pharmacological**	Primary microglial cells Mixed glial cell culture	LPS	Antidepressant activity of amitriptyline and nortriptyline	The anti-inflammatory activity of these drugs partially explained the multifactorial pathogenesis of depression, including neuroinflammation	Obuchowicz et al., 2006
	Primary microglial cells BV-2 mouse microglia cells	LPS	Antidepressant activity of fluoxetine	The therapeutic efficacy of fluoxetine was partially due to modulation of microglial activation	Liu et al., 2011
	N9 mouse microglial cells	LPS+ATP	Antidepressant activity of melatonin	Inhibition of microglial inflammatory activation was crucial for antidepressant activity of melatonin	Arioz et al., 2019
	Primary microglial cell culture	HMGB1/TNF-α	Antidepressant activity of arctigenin	Targeting microglial inflammatory activation provided a therapeutic avenue for treating depression	Xu et al., 2020

### Findings in Human Depression Studies

Although increased levels of proinflammatory cytokines, including IL-1β, IL-6, and TNF-α, in the plasma and CSF of depressed patients, have been reported, there still lies a big question mark on neuroinflammatory markers and microglial activation status (Martinez et al., 2012; Himmerich et al., 2019). The elevated translocator protein (TSPO) binding assessed by positron emission tomography studies in various brain regions of depressed patients backs microglial activation (Setiawan et al., 2015; Owen et al., 2017). Also, studies using other markers of microglial activation, such as Iba-1 or quinolinic acid, have found increased microglial reactivity in depression, whereas no difference between the density of major histocompatibility complex (HLA)-immunoreactive microglia in post-mortem brain samples of depressed subjects (Hamidi et al., 2004; Snijders et al., 2020). A recent study using single-cell high-dimensional mass cytometry (CyTOF) examined microglia from post-mortem MDD samples from different brain regions and found increased markers of homeostatic microglia, including transmembrane protein (TMEM)119 and P2Y12 in MDD compared to controls, which is in clear contrast with what has been found in animal studies (Bottcher et al., 2020). Gene expression analysis of microglia isolated from animal models of depression clearly showed enhanced inflammatory markers, including CD11b, CD45, and TLR4 (Wohleb et al., 2011; Lehmann et al., 2016). Moreover, gene expression profiling of post-mortem frontal lobe tissue from patients with MDD did not show any difference in the expression of IL-6 or TNF-α (Shelton et al., 2011). Furthermore, no differential expression of IL-6, IL-1β, or TNF-α mRNAs was found in post-mortem brain tissue of MDD cases (Bottcher et al., 2020).

It is not possible to mimic all the pathological features of human depression in animal models, owing to its multifactorial pathology involving epigenetic and genetic factors, multiple body systems working in conjunction, and subjectivity of symptoms. Yet, these models have provided useful insights into the neuroinflammatory mechanism of depression. Given that the role of neuroinflammation in human depression is yet not clear, the results of *in vivo* depression studies appear to be missing pieces of the puzzle of depression pathology (Nettis et al., 2021).

## Targeting Microglia and Neuroinflammation in Depression

Recent literature highlights the crucial role of brain immune cells in depression pathology and any modality that can modulate the activity of these cells or reduce neuroinflammation, thereby bearing the potential to treat depressive symptoms. Supporting this notion, beneficial effects of anti-inflammatory drugs have been observed in MDD patients (Muller et al., 2006; Abbasi et al., 2012; Kobayashi et al., 2013; Majd et al., 2015; Cao et al., 2020; Nettis et al., 2021). Clinical trials using non-steroidal anti-inflammatory drugs in depressed patients have reported promising results, with increased remission rates in patients when used in combination with conventional antidepressant drugs (Abbasi et al., 2012; Cao et al., 2020). A tetracycline antibiotic, minocycline, an inhibitor of microglial inflammatory activation, has also shown promising antidepressant activity in treatment-resistant depression patients (Kobayashi et al., 2013; Nettis et al., 2021). The antidepressant effects of minocycline were independent of changes in peripheral inflammatory biomarkers, reflecting the possible decrease in central inflammation (Nettis et al., 2021).

Mounting evidence also suggests the protective role of targeting neuroinflammation in *in vivo* models of depression. Pharmacological inhibition of caspase-1, which converts IL-1β to its mature form, alleviated the depressive phenotype in preclinical models by modulating neuroinflammatory pathways and stabilizing the surface expression of glutamate receptors (Li et al., 2018). Microglial activation was inhibited by pharmacological treatment with minocycline in a variety of stress models, reducing the increase of proinflammatory cytokines (Hinwood et al., 2012; Kreisel et al., 2014). Furthermore, minocycline attenuated stress-associated deficits in cognitive memory tasks, including the Morris water maze and Barnes maze, as well as depressive-like and anxiety-like behaviors, such as reduced social interaction, sucrose preference, and open field exploration (Hinwood et al., 2012). Moreover, pharmacological inhibition of microglial ATP-gated purinergic P2X7 receptor, activation of which leads to the maturation of IL-1β, also suppressed the development of depressive behavior in rodents subjected to CUMS (Bhattacharya et al., 2018). Adipose-derived mesenchymal stem cells also produced antidepressant effects by modulating microglial phenotype, suppressing TLR4/NF-κB signaling pathways, and upregulating antioxidant pathways in mice subjected to CUMS (Huang et al., 2020). Anesthetic ketamine, which has antidepressant potential, is also known to suppress inflammatory pathways (Abdallah et al., 2020). It has been recently demonstrated in CUMS model that ketamine suppressed microglial activation and NLRP1 inflammasome pathway, exerting antidepressant effects (Aricioglu et al., 2020). These preclinical studies suggest that targeting neuroinflammation appears to be a promising therapeutic approach.

## Conclusion and Prospects

Owing to complex neurobiology and genetic variability, depression cannot be fully mimicked in animal models. However, many molecular insights can be gained from these models to identify therapeutic targets for depression. The heightened role of neuroinflammatory cascades observed in animal models of depression and the efficacy of anti-inflammatory treatment in decreasing depressive behavior pinpoint the role of neuroinflammation in the neurobiology of depression. Moreover, the inefficacy of classical antidepressant drugs partly explains the unappreciated role of neuroinflammation in depressive disorders and paves a path for targeting neuroinflammation to treat depression.

## Author Contributions

RA conducted the literature review, formulated, and wrote the manuscript. KS edited the manuscript and was involved in all aspects of manuscript preparation. Both authors contributed to the article and approved the submitted version.

## Conflict of Interest

The authors declare that the research was conducted in the absence of any commercial or financial relationships that could be construed as a potential conflict of interest.
